# Probing and manipulating the gut microbiome with chemistry and chemical tools

**DOI:** 10.1017/gmb.2025.4

**Published:** 2025-04-14

**Authors:** Pavan K. Mantravadi, Basavaraj S. Kovi, Sabbasani Rajasekhara Reddy, Ganesh Pandian Namasivayam, Karunakaran Kalesh, Anutthaman Parthasarathy

**Affiliations:** 1 CMC and Analytical, Cytokinetics, South San Francisco, CA, USA; 2 Institute for Integrated Cell-Material Sciences (ICeMS), Kyoto University, Kyoto, Japan; 3 Department of Chemistry, School of Advanced Sciences, Vellore Institute of Technology (VIT), Vellore, India; 4 School of Health and Life Sciences, Teesside University, Middlesbrough, UK; 5 National Horizons Centre, Darlington, UK; 6 The School of Chemistry and Biosciences, University of Bradford, Bradford, UK

**Keywords:** gut microbiome, chemistry, prebiotics, conjugation inhibitors, chemical probes

## Abstract

The human gut microbiome represents an extended “second genome” harbouring about 10^15^ microbes containing >100 times the number of genes as the host. States of health and disease are largely mediated by host–microbial metabolic interplay, and the microbiome composition also underlies the differential responses to chemotherapeutic agents between people. Chemical information will be the key to tackle this complexity and discover specific gut microbiome metabolism for creating more personalised interventions. Additionally, rising antibiotic resistance and growing awareness of gut microbiome effects are creating a need for non-microbicidal therapeutic interventions. We classify chemical interventions for the gut microbiome into categories like molecular decoys, bacterial conjugation inhibitors, colonisation resistance-stimulating molecules, “prebiotics” to promote the growth of beneficial microbes, and inhibitors of specific gut microbial enzymes. Moreover, small molecule probes, including click chemistry probes, artificial substrates for assaying gut bacterial enzymes and receptor agonists/antagonists, which engage host receptors interacting with the microbiome, are some other promising developments in the expanding chemical toolkit for probing and modulating the gut microbiome. This review explicitly excludes “biologics” such as probiotics, bacteriophages, and CRISPR to concentrate on chemistry and chemical tools like chemoproteomics in the gut-microbiome context.

## Graphical abstract

**Figure:**
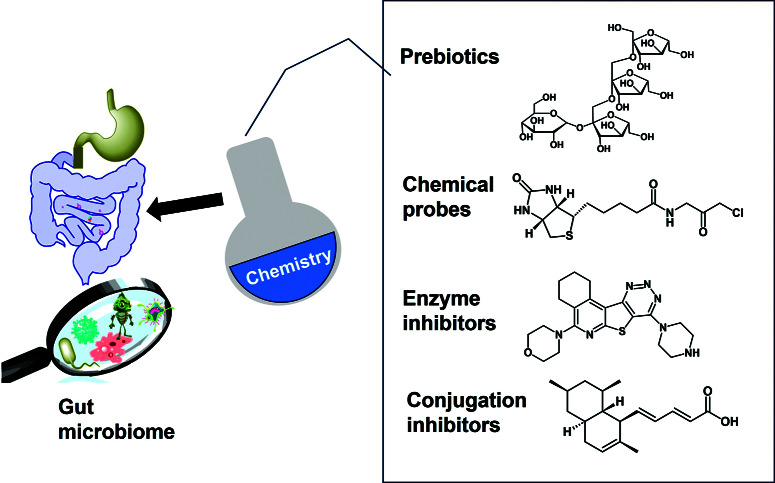

## Introduction

There are about 10^13^–10^15^ symbiotic microbes residing inside and on the surface of a human being which collectively constitute the human microbiome (Turnbaugh et al., 2007). The microbiome plays a significant role in lifelong host health (Rackaityte and Lynch, 2020) and underlies a considerable proportion of the individual differences in drug metabolism (Zimmermann et al., 2019). Therefore, modulating the human microbiomes has triggered the interest of both academia and industry, and several interventions have been designed to either preserve or rebuild the function of the microbiome. In the period 2015–2018, over 80 microbiome modulators entered the preclinical phase, while 15 were in phase I trials, 5 in phase II, and 6 in phase III, according to the Pharmaprojects 2018 Microbiome Whitepaper (Ltd., I. U, 2018). The same report details that as of 2018, 10 modulators were in the pipeline for metabolic disorders, 21 for gastrointestinal disorders, and 24 for infectious diseases.

The gut (gastrointestinal system) harbours the most extensive human microbiome, which is critical for host metabolic and immune functions (Shreiner et al., 2015). Further, a healthy microbiome also prevents pathogens from colonising the gut, a phenomenon known as colonisation resistance (CR) (Ducarmon et al., 2019). The gut also contains the largest surface where immune system activity occurs inside the human body (Kraehenbuhl and Neutra, 1992) and the development of the immune system itself is a delicate dance of balancing the host versus the gut microbes (Randall and Mebius, 2014). The gut connects to various distal organs via two-way signalling and therefore, the gut microbiome (GM) maintains far more than just gut health (Schroeder and Bäckhed, 2016). GM dysfunction is implicated in the development of infections, gastrointestinal cancers as well as liver, respiratory, neurological, cardiac, metabolic, and autoimmune diseases (Wang et al., 2017).

Antibiotics, in particular, cause deleterious changes to the function of the GM (Becattini et al., 2016) and therefore preserving/restoring those functions is important. The antimicrobial resistance crisis has also led to a search for less indiscriminate therapeutics, which are GM friendly (Patangia et al., 2022). Kang et al. showed that gut bacteria such as *Clostridium scindens* and *Clostridium sordellii* which perform 7α-dehydroxylation of bile salts, also produced endogenous narrow-spectrum antibiotics derived from tryptophan, such as turbomycin A and 1-acetyl-β-carboline which inhibit *Clostridioides difficile* (Kang et al., 2019). Indole-3-propionic acid (IPA), another tryptophan metabolite that is produced by *Clostridium sporogenes*, inhibits a variety of mycobacteria, including drug-resistant *Mycobacterium tuberculosis* (Negatu et al., 2020). IPA inhibited *M. tuberculosis* both *in vitro* and when administered in mice models via oral and intravenous routes (where it showed a seven-fold bacterial load reduction in the spleen (Negatu et al., 2018, 2020)). GM-derived IPA can bind and powerfully induce the aryl hydrocarbon receptor (AHR; a major regulator of both innate and adaptive immunity) and therefore modulate the susceptibility to *M. tuberculosis* (Negatu et al., 2020). The recovery of IPA in the serum (Negatu et al., 2020) and the existence of the gut-lung (Schroeder and Bäckhed, 2016) and gut-spleen (Barrea et al., 2017) axes explains how the GM can influence both lung and immune function remotely.

Endogenous narrow-spectrum peptide antibiotics with more complicated structures like bacteriocins also exist (Rea et al., 2010) and could become available for research via solid-phase peptide synthesis since synthetic methods for cyclic peptides are rapidly improving (Bédard and Biron, 2018). Drug delivery targeted to different gut compartments (Hua, 2020) is already a burgeoning field. Therefore, chemically synthesised narrow-spectrum antibiotics could, in the near future, be delivered to specific gut compartments for directly or indirectly influencing the susceptibility and host-colonisation ability of major pathogens such as *M. tuberculosis* (Negatu et al., 2020) and *C. difficile* (Kang et al., 2019) as well as modulating host immunity, to prevent infections or aid recovery from infections.

Direct chemical manipulation of the GM has been the most challenging to perform in the absence of prior knowledge of the targets. However, in a pioneering study, Chen et al. devised an *in vitro* screening protocol and were able to use the cyclic d,l-α-peptides they identified via screening to change GM induced by a Western diet into one reflecting a low-fat diet (Chen et al., 2020). This not only ameliorated atherosclerosis in mice, but also adjusted the levels of pro-inflammatory cytokines, short-chain fatty acids (SCFA), and bile acids (BAs) to healthy levels, while improving gut barrier integrity and T-cell function. They described their approach as “directed remodelling,” implying a deliberate manipulation of the GM in a predetermined manner from one state to another.

Research is moving away from largely cataloguing microbial strains to examining and understanding the molecular basis of the GM’s influence on human health (Rackaityte and Lynch, 2020). Therefore, we argue that chemistry and chemical information will play an important part in unravelling GM interactions and manipulating the GM to promote health. With this in mind, we focus on the roles of chemistry and chemoproteomics, while excluding “biologics” strategies such as probiotics, bacteriophages, and CRISPR. Narrow-spectrum antibiotics and directed chemical remodelling are only two recent examples of the potential of chemistry in the GM story. Whether preparing prebiotics, inhibiting bacterial conjugation in the gut, stimulating CR, probing GM-host interactions, or altering the GM composition to promote host health, the versatile toolkit of chemistry offers abundant opportunities to explore and modulate the GM.

## Molecules that preserve/restore the GM

**Figure 1.**
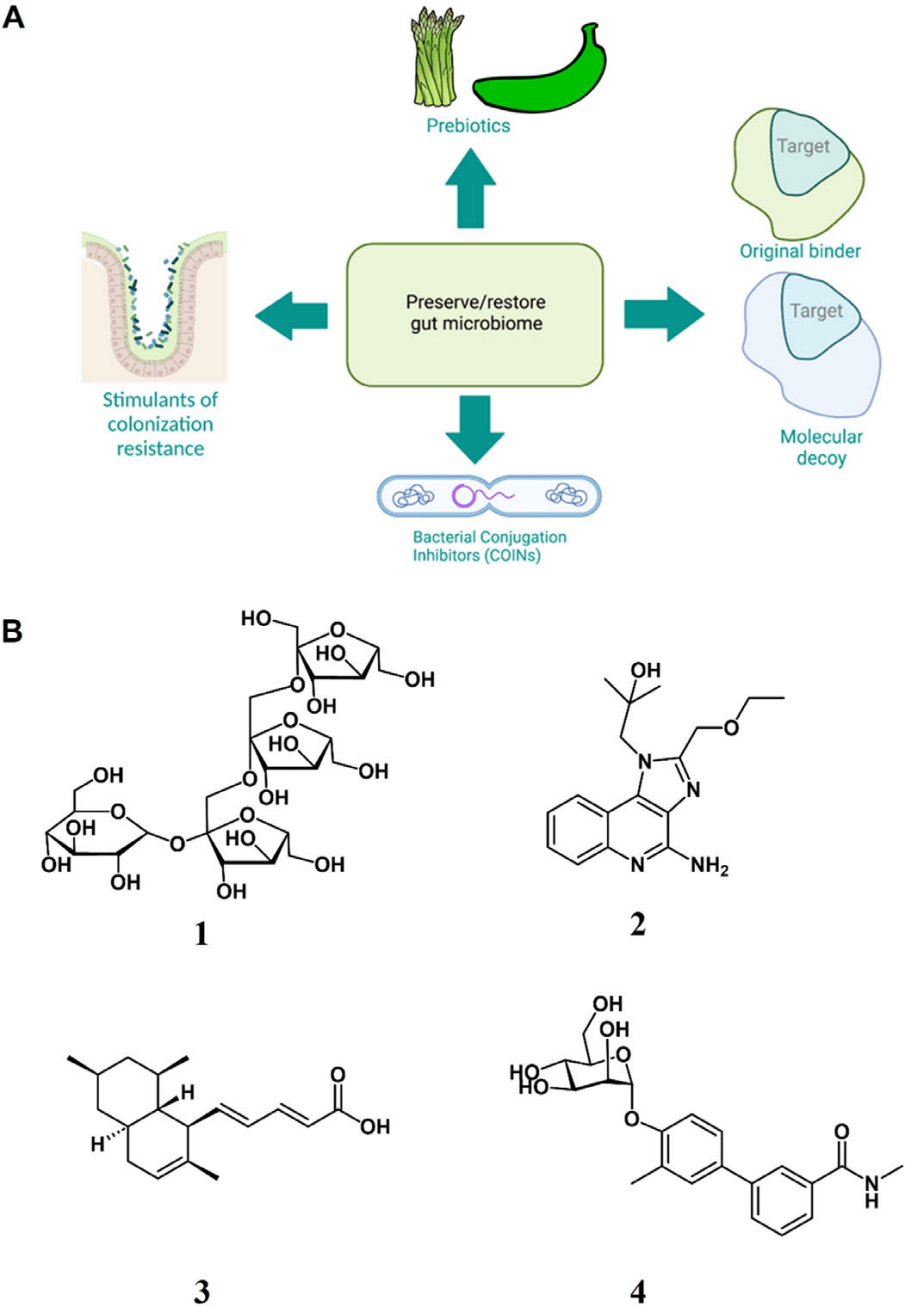
(A) Functional classification of molecules to preserve/restore the gut microbiome. (B) Chemical diversity of molecules with microbiome preserving/restoring functions; 1 = General structure of inulins (endogenous prebiotic), 2 = resiquimod or R848 (synthetic stimulant of colonisation resistance); 3 = tanzawaic acid B or TZA-B (natural product colonisation inhibitor); and 4 = a mannoside (mannose-containing decoy for urinary pathogens which preserves the gut microbiota).

These are classified based on their mode of action as shown in Figure 1A and some example chemical structures are shown in Figure 1B.

### Prebiotics

Prebiotics are selectively fermented ingredients that trigger specific changes in the microbiome composition and activity to promote host health (Gibson et al., 2004). Safely administering live microbes and establishing their colonisation in the gut is difficult and faces regulatory hurdles, making small molecule interventions more attractive (Cully, 2019). Small molecules, especially endogenous metabolites can accumulate to high concentrations with negligible toxicity, remain stable in the systemic circulation, and obey the principles of pharmacokinetics. The major prebiotics are human milk oligosaccharides (HMOs), inulins (**1** in Figure 1B), fructose oligosaccharides (FOS), xylooligosaccharides (XOS), mannan oligosaccharides (MOS), and galactooligosaccharides, which are polymers/oligomers of glucose, fructose, mannose, fucose, galactose, sialic acid, xylose, uronic acid, and arabinofuranose units linked together with β2, β3, and β4 linkages (Enam and Mansell, 2019).

Developments in chemical synthesis are bringing the goal of complex carbohydrate assembly closer. Difficulties arise mainly from (1) the need to selectively protect and deprotect monosaccharides and (2) regioselectivity and stereoselectivity. Improved glycosylation strategies have been reported, which enables glycosyl donors to react in a specific order, yielding a single oligosaccharide product (Vohra et al., 2008). Automated glycan assembly (AGA) currently enables access to a maximum length of 100, while convergent block coupling of 30- and 31-mer oligosaccharide fragments made by AGA was used to make a multiple-branched 151-mer polymannoside (Joseph et al., 2020).

Enzymatic and chemoenzymatic processes offer better regioselectivity and stereoselectivity, along with fewer steps in the synthesis, which makes them faster and more cost effective (Li et al., 2019). For example, the HMO 2′-fucosyllactose (2′FL) has been synthesised in engineered *Escherichia coli* strains (Baumgärtner et al., 2013). One-pot multi-enzyme synthesis has been reported which employs glycosyltransferases to synthesise sialyl- and fucosyl-derivatives (Chen et al., 2015). Sialylated HMOs with high regioselectivity and stereoselectivity have been synthesised using a chemoenzymatic strategy, whereby automated solid-phase synthesis of the glycan backbone was followed by α-(2,3)-sialyltransferase treatment (Fair et al., 2015). Interest in sustainable chemical feedstocks has led to method development for the conversion of lignocellulose biomass into valuable prebiotics such as XOS (Poletto et al., 2020).

Prebiotics can have synergistic interactions with approved drugs. Konjac MOS from the plant *Amorphophallus konjac* are prebiotics containing β-D-mannose and β-D-glucose residues linked by one to four linkages (Liu et al., 2015). The combined administration of the drug metformin and konjac MOS mitigates insulin resistance and glucose tolerance, while also improving islet and hepatic tissue function (Zheng et al., 2018). The beneficial effects were correlated with the reduced abundance of the Rikenellaceae family and the Clostridiales order, with an increased relative abundance of *Bifidobacterium pseudolongum*, *Akkermansia muciniphila*, and OTU05945 of family S24–7 (Zheng et al., 2018). Further studies focussing on prebiotic-drug interactions could lead to more targeted application of prebiotics in combination with approved drugs to mitigate the impact of specific diseases.

### Stimulants of CR

CR is a mechanism by which the gut microbiota protects itself against the incursion and establishment of largely harmful microorganisms. This protection can be accomplished by several routes, such as antimicrobial secretion, nutrient limitation, and stimulation of gut barrier integrity and the action of bacteriophages (Ducarmon et al., 2019). Disturbances to the gut resulting from the use of antibiotics, other drugs, or inflammation can reduce CR, allowing enteric pathogens such as *C. difficile*, *Salmonella enterica* serovar Typhimurium, *E. coli*, *Shigella flexneri*, *Campylobacter jejuni*, *Vibrio cholerae*, *Yersinia enterocolitica*, and *Listeria monocytogenes*, to colonise the niches vacated by microbiome disruption (Sorbara and Pamer, 2019). Both endogenous molecules such as SCFA and tryptophan metabolites produced by the GM and exogenous synthetic small molecules can restore CR function. Synthetic molecules are beginning to be used in efforts to stimulate CR following disturbances to the GM, for example, after antibiotic administration. For example, vancomycin-resistant enterococci (VRE) flourish when CR is compromised following antibiotic treatment. A synthetic molecule, resiquimod or R848 (**2** in Figure 1B), binds to a Toll-like receptor 7 that stimulates innate immune defences, leading to the restoration of CR against VRE by triggering the expression of the antimicrobial peptide Reg3γ (Abt et al., 2016). R848 can be taken orally and induces the secretion of interleukins IL-23 and IL-22.

### Bacterial conjugation inhibitors

Antibiotic resistance is spread by several mechanisms, including horizontal gene transfer mediated by plasmids. Analysis of *Bacteroidetes* strains sharing the intestinal niches of specific individual humans, demonstrated the extensive occurrence of horizontal gene transfer among those strains. In this case, the genetic elements exchanged coded for orphan DNA methylases, fimbriae synthesis proteins, novel metabolic enzymes, antibiotics, and proposed type VI secretion systems (T6SS) (Coyne et al., 2014). More recent studies have recorded extensive plasmid exchange in the gut environment using CRISPR-Cas spacer acquisition analysis in an *E. coli* strain (Munck et al., 2020). Unlike earlier studies that relied on phenotypic markers or post-transfer replication to detect mobile genetic elements, the spacer acquisition analysis reveals plasmid transfer in real time, and the results showed that the IncX plasmid type was most frequently transferred (Munck et al., 2020). Therefore, inhibiting bacterial conjugation in a bacteria-dense environment could enable the host to mitigate antibiotic-resistant infections. In general, resident bacteria in the healthy GM may be able to suppress the evolution of antibiotic resistance *in vivo.* However, the wide distribution of plasmid-borne resistance in the environment is well known and exposure to them might be common. Moreover, gut inflammation boosts plasmid transfer between pathogenic and commensal Enterobacteriaceae (Stecher et al., 2012). Therefore, inhibiting plasmid transfer in the gut is expected to promote host health, and conjugation inhibitors (COINs) are unlikely to disturb the GM composition, unlike conventional antibiotics. We describe a few known COINS, but some need to be further specifically tested in the gut environment.

Early studies to identify COINs unearthed many unspecific molecules that affected DNA replication or growth (Cabezón et al., 2017). Plant phenolics seems to be a good source of COINS and have yielded two molecules that specifically inhibited bacterial conjugation, namely rottlerin and 8-cinnamoyl-5,7-dihydroxy-2,2,6-trimethylchromene (Oyedemi et al., 2016). Screening of a library of over 12,000 NPs (NatChem library) based on high-throughput whole-cell-based assays enabled the discrimination between true COINS and false “hits” which merely affected cell growth, leading to the discovery of the COIN dehydrocrepnynic acid (DHCA) (Fernandez-Lopez et al., 2005). DHCA belongs to the chemical family of unsaturated fatty acids (UFAs), which is generally a good source of COINS. DHCA is derived from a tropical seed, and its supply is limited. However, it was used as the starting point for the synthesis of other COINS, particularly 2-hexadecynoic acid (2-HDA) and other 2-alkynoic fatty acids (2-AFAs), which specifically inhibited the transfer of a range of plasmids, including the common and highly infective IncF, in various bacteria (Getino et al., 2015). 2-HAD was later reported to prevent bacterial conjugation in the mouse gut (Palencia-Gándara et al., 2021). A series of UFA NPs called tanzawaic acids were discovered (tanzawaic acid B or TZA-B is depicted as (**3** in Figure 1B); they mainly inhibited conjugation by the IncW- and IncFII-based plasmids. Other plasmids classified under the IncFI, IncI, IncL/M, IncX, and IncH incompatibility groups were less affected, whereas the IncN and IncP plasmids were unaffected (Getino et al., 2016).

Conjugation is driven by the type 4 secretion system, whose architecture is conserved in most bacteria, and contains the pilus, the core channel complex, the inner membrane platform, and the ATPases that provide energy for substrate transport and pilus biogenesis (Cabezón et al., 2015). Nicking the DNA to relax the plasmid, DNA transfer to the secretion channel, the transfer of pilin molecules during pilus biogenesis, and pilus biogenesis are performed by four distinct ATP-ase enzymes, among which carboxylic acid COINS were shown to target the last step (TrwD protein). Based on structural and computational data, the UFAs and AFAs were suggested to bind at the end of the N-terminal domain as well as at the beginning of the linker region that connects the N-terminal and C-terminal domains, likely hindering the swapping movements of the domains needed for the catalytic cycle (Ripoll-Rozada et al., 2016).

### Molecular decoys

These molecules bind enteric pathogens and stimulate their elimination from the gastrointestinal tract. This binding is thought to “fool” pathogens by mimicking the receptors used by them to attach to the gut epithelia in the lower gastrointestinal tract. The global enteric diseases is substantial and cases may number in the hundreds of millions annually. HMOs act as soluble decoys for receptors and block the binding of enteric pathogens. Rotavirus infection is prevented most effectively by the HMO 2′FL, although several other HMOs also have similar inhibitory effects (Laucirica et al., 2017). *C. jejuni* infects the mammalian gut and causes diarrhoea and sometimes also motor neuron paralysis. The infection is initiated by the bacterium binding to the fucosylated intestinal H(O) antigen (Fuc alpha 1, 2Gal beta 1, 4GlcNAc). However, FOS in human milk can act as decoys by binding to the pathogen, thereby preventing infection (Ruiz-Palacios et al., 2003).

Uropathogenic *E. coli* (UPEC) uses the extracellular appendages called Type 1 pili to colonise the intestine by binding a mannosylated host receptor; the Type 1 pili are also essential for colonisation and infection in the bladder. Mannosides (**4** in Figure 1B) are small-molecule drugs bearing mannose group(s) that act as decoys by mimicking the mannosylated receptor and can clear both bladder and intestinal UPEC upon oral administration in mouse models, leaving the GM largely intact (Spaulding et al., 2018). The decoy approach has been further extended to combat cholera, and in this case, nanotechnology is also employed. The *V. cholerae* toxin binds to the host receptor monosialotetrahexosylganglioside (GM1), and coating GM1 on the surface of polymeric nanoparticles was enough to reduce cyclic-AMP production in epithelia and fluid responses to live *V. cholerae* in both cell cultures and a mouse infection model (Das et al., 2018). The modulation of disease via molecular mimicry extends to non-sugar molecules, such as metalloenzymes allows for the manipulation of the gut chemical environment using synthetic catalysts. A metalloporphyrin mimic of the enzyme superoxide dismutase could reduce lipid peroxidation levels and thereby shielded epithelial cells from damage in rats injected with the common antigen bacterial lipopolysaccharide (LPS) (Das et al., 2018).

## Chemical probes of the GM

The majority of recent chemistry-oriented studies did not deal with direct chemical manipulation of the GM but focussed on probing the GM using bio-orthogonal strategies such as alkyne-cycloazide addition, Staudinger ligation, and tetrazine ligation to create “chemical reporters” (Zhang et al., 2020). Bacterial surface glycans, peptidoglycans (PGNs), lipopolysaccharides, capsular polysaccharides (CPSs), glycoproteins, lipids, and other molecules, such as BAs have been labelled (Zhang et al., 2020). In addition to such surface targeting, protein function may be probed by activity-based protein profiling (ABPP), which involves small molecules reacting with mechanistically related enzymes (Berger et al., 2004). In ABPP, the probe usually contains a reactive group and a tag. Microbiota-metabolite interactions as well microbiome composition and dynamics can be interrogated via ABPP, while chemoproteomics advances have made the detection of covalent probe-tagged proteins following the ABPP routine (Zhang et al., 2020).

### Fluorophores

**Figure 2.**
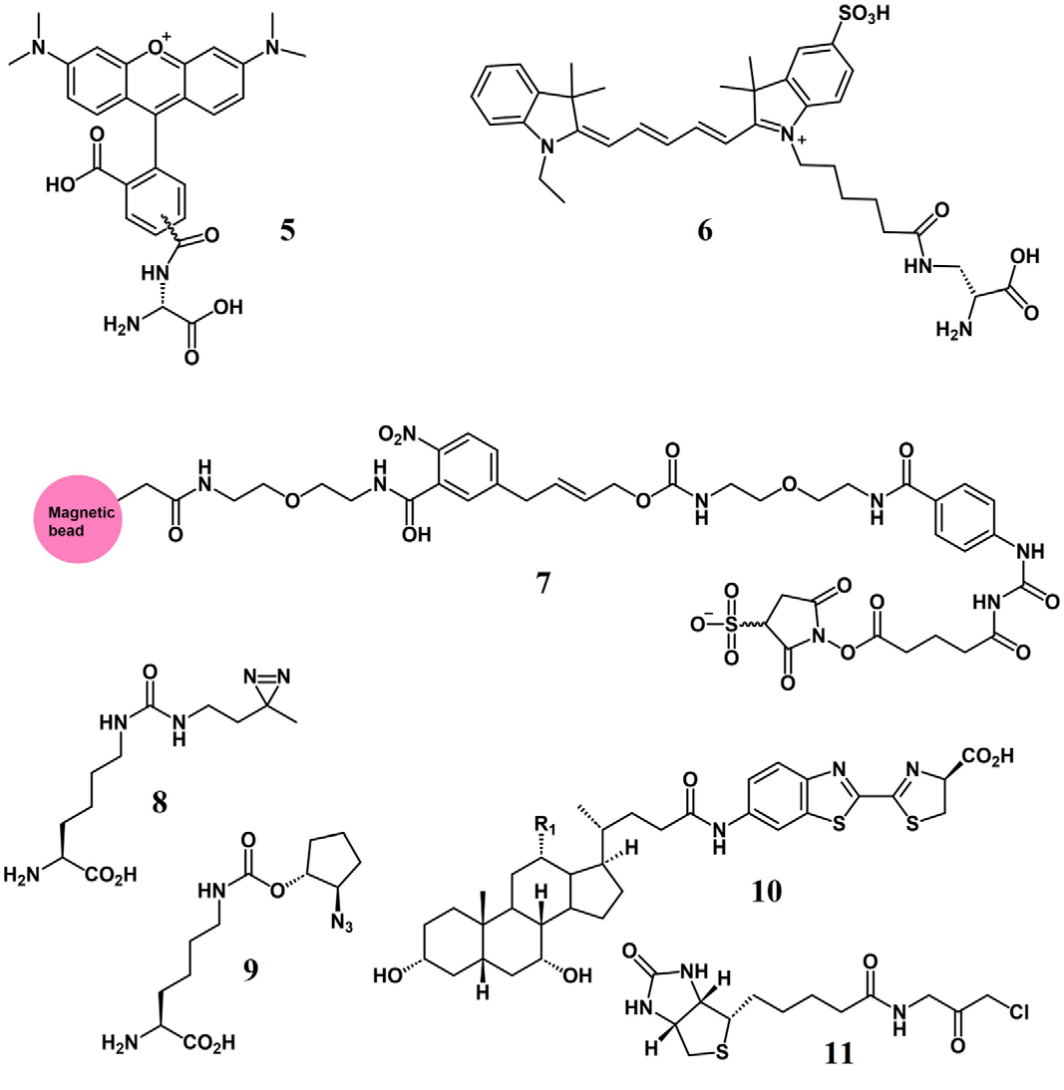
Examples of chemical probes used to interrogate the GM – D-amino acid-based fluorescent probes = TADA (**5**) and Cy5ADA (**6**); a multifunctional probe showing different parts shaded in distinct colours = amine directed probe based on sulpho-N-hydroxysuccinimide (**7**); photoactive unnatural amino acid probes = DiZPK (**8**) and ACPK (**9**); a cysteine-targeted probe = Biotin-Gly-CMK (**10**); bioluminescent bile acid-luciferin conjugates for bile salt hydrolase (BSH) activity = series of compounds with H or OH at the positions R1 and R2 (**11**).

The most common tools for probing the GM are fluorophores, which may be attached to different types of other chemical entities. Commensal anaerobic bacteria, including *Bacteroides fragilis* when fed azide-labelled sugars, which subsequently conjugated with alkyne-fluorophores via click chemistry, facilitate the imaging of bacteria in live mice (Geva-Zatorsky et al., 2015). Three different bacterial surface molecules from the GM, which interact with the host immune system, namely LPS, CPS, and PGN, can be tracked (Hatzenpichler et al., 2014), helping to dissect host–microbe interactions. Azide-bearing amino acids when fed to complex gut microbial communities showed that newly synthesised proteins could be visualised *in situ* (Hatzenpichler et al., 2014). Two D-amino acid-based fluorescent probes, TADA and Cy5ADA (5,6 in Figure 2), which get incorporated into bacterial PGN, have been instrumental in enabling live monitoring of GM growth and division patterns in mice (Lin et al., 2020). Probes based on D-amino acids are also being used to track the viabilities of bacteria in faecal transplants by using sequential tagging (Wang et al., 2019). In this approach, the bacteria are treated with a probe before the transplantation and then the recipient mice are fed a second probe following the transplantation. Therefore, the bacteria surviving the process show the emission for both probes, enabling the identification of viable bacteria in the transplant (Wang et al., 2019).

### Multifunctional selective probes

Direct extraction from human faecal samples and release under mild conditions is possible using multifunctional chemoselective probes (Garg et al., 2018), allowing for the analysis of femtomole levels of metabolites with enhanced sensitivity. Probe **7** in Figure 2 is anchored at one end to magnetic beads, linked by a spacer to a novel *p*-nitrocinnamyloxycarbonyl biorthogonal cleavage site, while the reactive site features an amine-selective sulpho-N-hydroxysuccinimide “warhead,” which reacts with metabolic amines (Garg et al., 2018). Since 2011, it has been possible to monitor enteric pathogens via the incorporation of the photoactive unnatural amino acids DiZPK and ACPK (**8, 9** in Figure 2) into specific pathogen proteins, which react to form cross links revealing the interactions between the modified protein and its client proteins (Lin et al., 2011). This approach is enabling the direct identification of proteins involved in pathogenesis and acid-stress defence mechanisms, which is quite challenging to perform with conventional methods.

### Simple reactive probes

Sphinganines are bioactive components of foods, but the GM also modifies them. The use of alkyne-tagged sphinganines allows for the identification sphinganine-utilising GM strains based on labelling followed by a cell sorting workflow (Lee et al., 2021). The subsequent sequencing of the sorted bacteria revealed that this metabolism is nearly exclusively performed by members of the *Bacteroides* (Lee et al., 2021). An activity-based probe, Biotin-Gly-CMK (**10** in Figure 2), has been used to differentiate between mice models harbouring “normal” human GM and “inflammatory bowel disease” (IBD) affected human GM, whereby a novel cysteine-reactive probe tagged several proteases and hydrolases in the IBD model, but not in the healthy controls (Mayers et al., 2017).

An elegant recent study by Nie et al. using a click chemistry strategy isolated and identified a previously unknown BA 3-succinylated cholic acid correlated with reduced progression of metabolic dysfunction-associated steatohepatitis in humans (Nie et al., 2024). Using this discovery, the authors were able to characterise an annotated β-lactamase in the GM member *Bacteroides uniformis* as the enzyme catalysing the 3-succinylation of CA (Mayers et al., 2017).

### Bioluminescent probes

Luciferin-based bioluminescent probes (**11** in Figure 2) have been employed to detect bile salt hydrolase (BSH) activity in a wide variety of sample environments including purified enzymes, bacterial cells, faecal slurries as well as non-invasive imaging in mice and humans (Khodakivskyi et al., 2021). BSH activity releases luciferin from the conjugated BA, which can be further assayed using luciferase. These BA-luciferin probes were useful in demonstrating the stimulatory effect of prebiotics on BSH activity and as diagnostic tests that non-invasively detect the clinical IBD status in human patients (Khodakivskyi et al., 2021).

## Modulating specific enzyme functions in the GM

Targeting specific enzymes among the thousands of proteins actively produced by the gut microbes is a viable strategy for microbiome modulation.

### Choline metabolism

**Figure 3.**
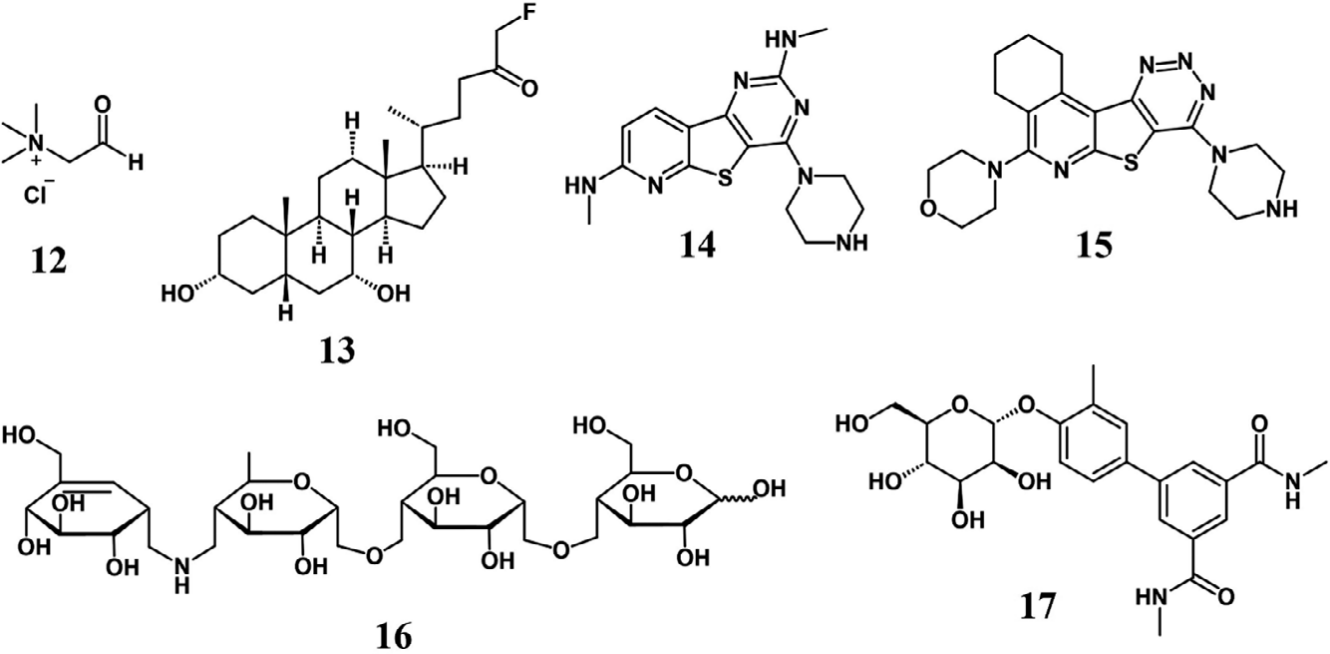
Specific enzyme inhibition can be a strategy to selectively manipulate the gut microbiome, and some inhibitors of gut bacterial enzymes are shown. **12** = betaine aldehyde, inhibits choline TMA-lyase (CutC); **13** = fluoromethyl ketone suicide inhibitor of bile salt hydrolase (BSH); **14**, **15** = piperazine-containing β-glucuronidase inhibitors; **16** = acarbose, inhibits starch and pullulan utilisation; and **17** = M4284 mannoside, inhibits FimH in uropathogenic *E. coli.*

A “chemically guided functional profiling” could be a strategy to uncover the presence of novel enzymes in the GM and subsequently, to modulate their function to achieve therapeutic effects. The conversion of choline into trimethylamine (TMA) by anaerobic gut bacteria is correlated with disease conditions in humans, and more specifically, the production of TMA in both isolated bacteria and complex communities can be inhibited by betaine aldehyde (**12** in Figure 3) (Orman et al., 2019). The identified target is GM choline TMA-lyase (CutC) and this opens up the scope for the development of other inhibitors.

### Bile salt metabolism

Bile salts have major effects on the physiology and virulence of *C. difficile.* When patients are restored to a *C. difficile*-resistant state, it is observed that the production of deoxycholate from cholate by 7α-dehydroxylating gut bacteria occurs (Savidge and Sorg, 2019). Broad-spectrum antibiotics block the production of secondary BAs and kill the 7α-dehydroxylating bacteria, thereby enabling *C. difficile* to colonise the gut (Savidge and Sorg, 2019). BSH enzymes expressed by the GM and bile salt metabolism affect the immune and metabolic processes via engaging host receptors. Therefore, inhibiting BSH enzymes would enable the dissection of the role of bile salts in host–microbe interactions. Screening a library of compounds, Adhikari et al. zeroed in on a covalent suicide inhibitor containing an α-fluoromethyl ketone moiety (**13** in Figure 3), which reacts with the active site cysteine of BSH enzymes, as way to globally modulate BSH and understand their physiological roles (Adhikari et al., 2020).

### Glucuronidase inhibitors

β-Glucuronidase (GUS) enzymes harboured by gut microbes can cause severe toxicity reactions to certain pharmaceuticals including cancer drugs; therefore, GUS inhibitors have been developed (**14, 15** in Figure 3) to ameliorate these toxic side effects. Pellock et al. reported the discovery of piperazine-based GUS inhibitors by combining chemical biology, protein structural data and mass spectrometry with cell-based assays (Pellock et al., 2018). Their GUS inhibitors interrupt the catalytic cycle of the enzyme and are substrate-dependent, binding to the catalytic intermediate by means of a piperazine-linked glucuronide. The inhibitor-glucuronide conjugates were detected by LC-MS (Santilli et al., 2018).

### Carbohydrate metabolism

The prospects for chemical precision editing of the GM are improving due to an expansion in the knowledge of its metabolism. GM diversity is promoted by the metabolism of complex plant polysaccharides. Selective manipulation of polysaccharide metabolism without microbicidal effects has been achieved using a small molecule inhibitor, acarbose (**16** in Figure 3), which abolished the ability of *Bacteroides thetaiotaomicron* and *B. fragilis* to utilise potato starch and pullulan by interfering with the starch utilisation system (). Shifting the GM metabolic activity selectively in this non-lethal fashion alleviated colitis. Until recently, it was not known if single bacterial species or a small community is needed to drive the degradation of any highly complex polysaccharide. The most complex polysaccharide characterised in the gut environment is rhamnogalacturonan-II, which is depolymerised by *B. thetaiotaomicron* with the cleavage of 20 out of its 21 distinct glycosidic bonds (Ndeh et al., 2017). Further analysis revealed that several previously unknown bacterial enzymes were responsible for the degradation of rhamnogalacturonan-II.

### Miscellaneous inhibitors

Zhu et al. showed that dysbiosis-linked gut inflammation caused by the expansion of facultative anaerobic Proteobacteria could be blocked via tungstate administration, which inhibits molybdenum-cofactor respiratory chain enzymes (Zhu et al., 2018). The GM composition was undisturbed when tungstate was administered under homeostatic conditions. Recurrent infections of the urinary tract caused by UPEC occur in 30–50% of patients even after antibiotic treatment. This persistence is linked to the type 1 pilus adhesin, FimH, which binds mannose and aids the colonisation of the bladder surface. Type 1 pili were also shown to aid UPEC colonisation in the gut, and the administration of the high affinity FimH inhibitor mannoside M4284 (**17** in Figure 3) reduced gut colonisation and urinary tract infection caused by genetically distinct UPEC isolates, without disrupting the GM composition (Spaulding et al., 2017).

## Chemoproteomics tools for GM studies

Over 1900 uncultured gut microbes were discovered in 2019 (Almeida et al., 2019), showing enormous potential for finding metabolic diversity in the GM. Metagenomics projects, including the Human Microbiome Project show that identification of the biochemical functions of genes encoding metabolic enzymes in the human GM accurately is fraught with difficulty. In a survey of 139 stool metagenomes, only around 30% of them could be assigned a gene ontology or enzyme commission annotation; of these annotations, 50% have previously unknown functions (Joice et al., 2014). Even in the case of enzymes/pathways that could be annotated, the gut microbiota contains many uncharacterised gene products detected in genomics/metagenomics analysis. Therefore, chemical information-based analyses (including analysis of chemical structure, chemical reactivity, and potential biological interaction partners) that predict potential GM metabolism and chemoproteomics methods are better placed to elucidate those “unknown” metabolic functions rather than purely metagenomics. Examples of the chemical information-based analysis include the design of gut-targeted drugs (Gil-Pichardo et al., 2023) and predictions of potential drug/xenobiotic metabolism in the GM (Malwe et al., 2023). Herein, however, we focus on some chemoproteomics/metabolomic tools developed for specific metabolite groups.

### Enzyme-based sulphated metabolome analysis

Sulphated compounds are derived from gut microbial transformation of dietary material and are related to disease states. Using an arylsulphatase enzyme to hydrolyse sulphated compounds and mass spectrometry-based metabolite analysis, Correia et al. have characterised and validated 235 sulphated metabolites in a single study, which were the products of gut microbiota and subsequent host transformations and discovered 11 previously unknown sulphated metabolites (Correia et al., 2020). The metabolites reported in this study could form the basis of classification of human subjects as harbouring high or low sulphate metabolising microbiota for future cohort studies. Further, the arylsulphatase-based method may be useful for discovering novel sulphated metabolites.

### BSH and BA-based chemoproteomics

As mentioned, BAs are secreted by the liver and are further converted into secondary BAs by the action of the GM. The latter participate in several processes, including the metabolism of glucose and lipids, and immune homeostasis. The key reaction of secondary BA biosynthesis is catalysed by BSH. BSH are bacterial cysteine hydrolases whose activity precedes other kinds of BA transformations (Devlin and Fischbach, 2015). Parasar et al. developed a strategy based on the covalent labelling of the active site cysteine using a substrate analogue (Parasar et al., 2019). When the substrate analogue is covalently bound, biorthogonal click chemistry could be applied to attach either a fluorescent contrast agent or a biotin affinity tag to the enzyme-bound analogue. In the first case, in situ imaging could be performed following gel electrophoresis, and in the second case, affinity purification was performed using streptavidin (the samples were subsequently analysed using proteomics).

While the expression of metagenomic fragments in well-studied model microbes showed that at least three distinct phyla possess BSH activities in the GM (Jones et al., 2008), genome-based strategies suffer from the issues of potential toxicity, incomplete coverage, incomplete BGC expression, unintended changes in enzyme levels and tissue localisation, all of which led to deviations from the physiologically relevant states of the BSH enzymes. By comparison, the covalent modification of the active sites of BSH enzymes coupled with proteomics has avoided many of the pitfalls associated with genome-based methods and enabled the direct identification of these enzymes.

While BAs promote CR, little was known about the target proteins affected in the gut pathogens inhibited by BA action. Photoaffinity probes based on chenodeoxycholic acid (CDCA) were able to crosslink many host and pathogen proteins in *Salmonella enterica* serovar Typhimurium infection models, of which direct protein inhibition by CDCA probes was reported for HilD, a key regulator of Salmonella pathogenesis and virulence (Yang et al., 2023). Chemical proteomics and photoaffinity labelling based on lithocholic acid were also used to identify a previously unknown BA-binding transcription factor called BapR in *C. difficile (*Forster et al., *2022).*

### Direct lysine-acylation chemoproteomics

In a 2022 report, abundant post-translational lysine-acylation by reactive acyl-CoA species was discovered, whereby the acyl motifs found on several differentially expressed proteins corresponded to the metabolism of specific carboxylic acids in syntrophic bacteria (Muroski et al., 2022). The importance of cross-feeding in the gut environment, the abundance of SCFA and the ability to analyse the proteome for post-translational modifications without highly biased pre-enrichment, direct analysis of lysine acylation in the GM has good potential to shed light on metabolomic aspects.

### Vitamin affinity probe chemoproteomics

*Bacteroidetes* are one of the four major GM phylas; their genomes usually encode several B_12_-dependent enzymes, although they lack the ability of *de novo* cobamide synthesis (Shelton et al., 2019). It is therefore likely that they could harbour B_12_ transport proteins different at the sequence level from canonical *E. coli* counterparts. The use of B_12_-based affinity probes and subsequent application of chemoproteomics in *B. thetaiotaomicron* samples revealed the presence of proteins without previously unknown functions; one of these proteins, BtuH2, was shown to capture and transport B_12_ directly *in vitro* and responsible for gut fitness of these bacteria in gnotobiotic mice (Putnam et al., 2022).

## Modulating host receptors

The intestinal surface senses bacterial surface molecules and GM metabolites through several types of cell-surface receptors, and further effects are exerted by receptor protein complexes inside various types of gut cells. Here, we briefly consider only selected agonists/antagonists linked to the GM activity of a few cell-surface, nuclear, and peroxisome-linked receptors.

### Cell-surface receptors

**Figure 4.**
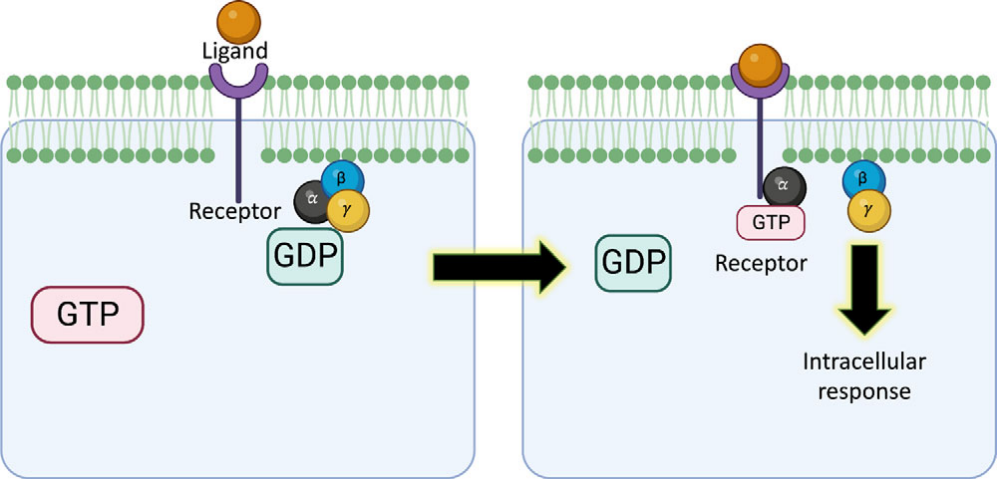
Molecular mechanism of G protein-coupled receptors on the cell surface. The ligand binds to the receptor protein, causing the G-protein subunits to disassemble and exchange bound GDP with GTP. The G-protein α-subunit is bound to the receptor, whereas the other subunits signal to other proteins involved in intracellular responses. GTP hydrolysis drives the dissociation of the α-subunit from the receptor and a return to the GDP-bound multi-subunit G-protein complex.

G protein-coupled receptors (GPCRs) are the largest membrane protein family in humans and sense their ligands through a mechanism outlined in Figure 4. GPCR complexes contain a transmembrane subunit (green in Figure 4) that binds a small molecule (ligand) at the cell surface, whereas a linked trimeric G-protein bound to GDP is located inside the cell. Once the ligand has been captured by the receptor subunit, then a conformational change occurs in the complex, allowing GTP to bind the trimeric G-protein, which usually dissociates, triggering an intracellular response via further downstream events. There are a variety of GPCRs in the gut for various microbial metabolites, such as SCFA (Husted et al., 2017), BAs (Lefebvre et al., 2009), and several other types of effectors (Cohen et al., 2015). Gut bacteria synthesise molecules such as commendamide, which mimic the human (endogenous) ligands of GPCRs (Kroeze et al., 2015).

A forward genetic screen (i.e., trying to identify genes leading to a phenotype) based on the Tango β-arrestin recruitment assay (PRESTO-Tango) was able to measure the activation processes of almost all the non-olfactory human GPCRs (Chen et al., 2019) and revealed several novel GPCR ligands such as l-phenylalanine secreted in the GM (Colosimo et al., 2019). Several other ligands that bind GPCRs (including in immune and nerve cells) such as phenylpropanoic acid, cadaverine, 9-10-methylenehexadecanoic acid, and 12-methyltetradecanoic acid were identified in a high-throughput screening of 241 GPCRs (Kovatcheva-Datchary et al., 2019), using seven gut microbes to represent a simplified human microbiome consortium (Subramanian et al., 2014; Kovatcheva-Datchary et al., 2019).

#### Free fatty acid receptors

SCFAs are sensed by specialised GPCRs called the free fatty acid receptor (FFAR), a family of cell surface receptors (Brown et al., 2003; Nilsson et al., 2003; Subramanian et al., 2014). FFAR2 and FFAR3 signalling links the GM and the β-cells in the pancreas and therefore are important targets in type-1 and type-2 diabetes (Nilsson et al., 2003; Priyadarshini et al., 2018). In pigs, the use of trans-glycosylated starches (TGS) led to downregulated FFAR2 via GM modulation, which decreased obesity (Priyadarshini et al., 2018). GM-derived SCFA and LPS also participate in the gut-lung immune axis because these molecules can travel to the lungs and modulate FFAR2/3 activity there (Husted et al., 2017).

#### Hydroxy carboxylic acid receptor

This is yet another class of GPCRs, which regulate immunity and energy homeostasis and sense hydroxycarboxylic acids. Most mammals have HCA1 that senses lactic acid, and HCA2 that senses 3-hydroxybutanoate and butyrate (Priyadarshini et al., 2021). Recently, a third hydroxy carboxylic acid receptor (HCAR) called HCA3 was detected in hominin genomes and described in humans; it senses and is potently activated by D-phenyllactic acid (Newman et al., 2018), which is produced as an antimicrobial by GM Lactobacilli. HCA2 is expressed in not only the intestinal epithelial cells, but also adipocytes, immune cells, hepatocytes, retinal epithelium, and Langerhans cells (Liu et al., 2021), suggesting involvement in communication between the gut and the fatty tissues, liver, eyes, and skin. It is implicated in pathological states such as intestinal inflammation and cancers, making it a possible therapeutic target in several diseases (Liu et al., 2021).

### Nuclear receptors

The major nuclear receptors in the gut are the AHR, the farnesoid X receptor (FXR), and the pregnane X receptor (PXR).

#### Aryl hydrocarbon receptor

This receptor is a transcription factor with a helix-lop-helix motif and senses compounds bearing an aromatic ring, such as indole/tryptophan compounds, polyphenols, flavonoids, and synthetic pollutants like dioxins and polycyclic aromatic hydrocarbons. It controls immunity at the gut barrier via the differentiation and inflammatory responses of innate and adaptive immune cells (Offermanns, 2017; Peters et al., 2019). GM tryptophan catabolism produces AHR ligands, such as indole-3-aldehyde, which stimulate intestinal immunity against *Candida albicans* colonisation via IL-22 (Li et al., 2021). Tryptophan metabolites also communicate bi-directionally between the GM and the brain (gut-brain axis) via the AHR (Shinde and McGaha, 2018). The natural dye indigo binds the AHR and induces the production of interleukins IL-10 and IL-22, which confers protection against high-fat diet (HFD)-induced insulin resistance and fatty liver disease in mice (Trikha and Lee, 2020). This was linked to specific increases in *Lactobacillus* cell counts and the elicitation of IL-22 secretion in the gut (Zelante et al., 2013). Intestinal inflammation can be modulated by AHR ligands such as oxazoles (Ma et al., 2020) and 6-formylindolo (3,2-b) carbazole (Ficz) (Lin et al., 2019).

#### Farnesoid X receptor

FXR is activated by BAs and is involved in lipid and glucose metabolism as well as energy homeostasis through the enterohepatic route (Iyer et al., 2018; Lamas et al., 2020). The antioxidant compound tempol leads to the accumulation of tauro-β-muricholic acid (T-β-MCA) in mice by blocking BSH enzymes in the Lactobacilli; T-β-MCA inhibits FXR signalling, consequently reducing obesity (Li and Chiang, 2014). Glycine-β-MCA prevents obesity, insulin resistance, and fatty liver disease in mice by decreasing the Firmicutes to Bacteroidetes ratio, leading to reduced SCFA levels (Li et al., 2013). BAs conjugated to the amino acids phenylalanine, tyrosine, and leucine are FXR agonists and are elevated in cystic fibrosis and IBD (Zhang et al., n.d.).

The BA derivative obeticholic acid (OCA) can reshape the small intestine microbiome in humans and mice via the FXR receptor (Quinn et al., 2020). These studies demonstrated the links between the GM, FXR and metabolic diseases and showed that FXR agonists could be promising anti-obesity leads via microbiome remodelling. In addition, OCA could also reduce the severity of *C. difficile* infection in mice fed an HFD by an FXR-mediated drop in primary BA levels, which decreases *C. difficile* spore germination (Friedman et al., 2018). Owing to the communication between the GM and the brain (the gut-brain axis), OCA can influence the GM-triggered microglial accumulation in the brain and ameliorate the anxiety associated with metabolic disease of treated mice (Jose et al., 2021). Small-molecule manipulation of the GM therefore enables the modulation of distant organs via the gut-brain, the gut-liver, the gut-heart, and the gut-lung axes.

#### Pregnane X receptor

PXR is implicated in the metabolism of xenobiotic compounds and is expressed in the vascular endothelium lining the blood vessels and is in direct contact with the serum (Wu et al., 2021). It is involved in innate immunity via the inflammasome and protection of the endothelia from oxidative damage (Wang et al., 2014). The natural product tanshinone IIA protects the endothelial cells from ROS damage via PXR activation (Zhu et al., 2017), while the GM metabolite indole-3-propionate (IPA) regulates PXR-dependent vasodilation (Pulakazhi Venu et al., 2019). Using IPA as a scaffold, Dvořák et al. synthesised a series of indole derivatives that were the first ever non-cytotoxic PXR agonists that reduced inflammation in mice (Dvořák et al., 2020), suggesting that GM metabolite mimicry might be a viable strategy to discover novel drugs with good efficacy and low toxicity.

### Peroxisome proliferator-activated receptors

Peroxisome proliferator-activated receptors (PPARs) are found throughout the gut tissue and have roles in fatty acid sensing, metabolism, and modulation of immunity; PPARα is crucial for fatty acid and branched chain amino acid catabolism in the mitochondria and peroxisomes (Grabacka et al., 2021), while PPARγ is important in innate immunity (Croasdell et al., 2015). Double agonists of both these receptors have been successful in animal models of *Citrobacter rodentium* and DSS-induced colitis of reducing tissue damage and bacterial loads leading to infection clearance and resolved inflammation compared with agonists of each receptor separately (Katkar et al., 2022). PPARα and γ activation has been reported for keto- and hydroxy-octadecanoic acid species, which were produced by *Lactiplantibacillus plantarum (*Goto et al., *2015).* Oleoylethanolamide, an endogenous PPAR ligand, can be administered exogenously in mice to shift the microbiota in the colon to a higher Bacteroidetes/Firmicutes ratio, with corresponding increases in *Bacteroides*, *Prevotella*, and *Parabacteroides* and decreases in *Bacillus* and *Lactobacillus* strains (Di Paola et al., 2018). The GM has also been modulated by synthetic agonists, such as fenofibrate, which led to increased SCFA levels in serum and tissues in mice fed HFDs (Wang et al., 2022). Dysbiosis induced by either high-fructose diets or HFD in mice could be remediated by the PPAR agonist Wy-16434, whereby the Bacteroidetes/Firmicutes ratio increased (reduced Proteobacteria and increased Actinobacteria) (Silva-Veiga et al., 2020; Silva-Veiga et al., 2022).

## Future directions and conclusions

As outlined in this article, approaches such as the inhibition of specific GM metabolism, the use of COINS, the prophylactic use of small-molecule determinants of CR, and GM metabolite mimicry could emerge as therapeutic avenues in GM modulation and precision medicine. Outside the coverage of this article, developments in canonical amino acid modification, biorthogonal chemistry, non-canonical amino acids, ribosome engineering, mass spectrometry, natural product databases, and machine learning have increased the scope of chemical and chemical information-based tools to interrogate GM-related metabolism and discover GM-related natural products. The emergence of chemical and informatics technologies alongside advances in deep sequencing (Liu et al., 2022a), improvement in technologies to cultivate “uncultivable microbes” (Liu et al., 2022b), and isolate GM-specific microbes via “culturomics” (Diakite et al., 2020) make it an exciting time to be a chemical biologist interested in GM research, with expanding opportunities for chemistry-based discovery and interventions to benefit human health.

## References

[r1] Abt MC, Buffie CG, Sušac B, Becattini S, Carter RA, Leiner I, Keith JW, Artis D, Osborne LC and Pamer EG (2016) TLR-7 activation enhances IL-22-mediated colonization resistance against vancomycin-resistant enterococcus. Science Translational Medicine 8(327), 327ra325. 10.1126/scitranslmed.aad6663. PMC4991618 26912904

[r2] Adhikari AA, Seegar TCM, Ficarro SB, McCurry MD, Ramachandran D, Yao L, Chaudhari SN, Ndousse-Fetter S, Banks AS, Marto JA, et al. (2020) Development of a covalent inhibitor of gut bacterial bile salt hydrolases. Nature Chemical Biology 16(3), 318–326. 10.1038/s41589-020-0467-3. 32042200 PMC7036035

[r3] Almeida A, Mitchell AL, Boland M, Forster SC, Gloor GB, Tarkowska A, Lawley TD and Finn RD (2019) A new genomic blueprint of the human gut microbiota. Nature 568(7753), 499–504. 10.1038/s41586-019-0965-1. 30745586 PMC6784870

[r4] Barrea L, Di Somma C, Muscogiuri G, Tarantino G, Tenore GC, Orio F, … Savastano S (2017) Nutrition, inflammation and liver-spleen axis. Critical Reviews in Food Science and Nutrition 58(18), 3141–3158. 10.1080/10408398.2017.1353479. 28799803

[r5] Baumgärtner F, Seitz L, Sprenger GA and Albermann C (2013) Construction of *Escherichia coli* strains with chromosomally integrated expression cassettes for the synthesis of 2′-fucosyllactose. Microbial Cell Factories 12, 40. 10.1186/1475-2859-12-40. 23635327 PMC3655002

[r6] Becattini S, Taur Y and Pamer EG (2016) Antibiotic-induced changes in the intestinal microbiota and disease. Trends in Molecular Medicine 22(6), 458–478. 10.1016/j.molmed.2016.04.003. 27178527 PMC4885777

[r7] Bédard F and Biron E (2018) Recent progress in the chemical synthesis of class II and S-glycosylated bacteriocins. Frontiers in Microbiology 9, 1048. 10.3389/fmicb.2018.01048. 29875754 PMC5974097

[r8] Berger AB, Vitorino PM and Bogyo M (2004) Activity-based protein profiling: applications to biomarker discovery, in vivo imaging and drug discovery. American Journal of Pharmacogenomics 4(6), 371–381. 10.2165/00129785-200404060-00004. 15651898

[r9] Brown AJ, Goldsworthy SM, Barnes AA, Eilert MM, Tcheang L, Daniels D, Muir AI, Wigglesworth MJ, Kinghorn I, Fraser NJ, et al. (2003) The Orphan G protein-coupled receptors GPR41 and GPR43 are activated by propionate and other short chain carboxylic acids. Journal of Biological Chemistry 278(13), 11312–11319. 10.1074/jbc.M211609200. 12496283

[r10] Cabezón E, de la Cruz F and Arechaga I (2017) Conjugation inhibitors and their potential use to prevent dissemination of antibiotic resistance genes in bacteria. Frontiers in Microbiology 8, 2329. 10.3389/fmicb.2017.02329. 29255449 PMC5723004

[r11] Cabezón E, Ripoll-Rozada J, Peña A, de la Cruz F and Arechaga I (2015) Towards an integrated model of bacterial conjugation. FEMS Microbiology Reviews 39(1), 81–95. 10.1111/1574-6976.12085. 25154632

[r12] Chen C, Zhang Y, Xue M, Liu X-w, Li Y, Chen X, Wang PG, Wang F and Cao H (2015) Sequential one-pot multienzyme (OPME) synthesis of lacto-N-neotetraose and its sialyl and fucosyl derivatives. Chemical Communications 51(36), 7689–7692. 10.1039/C5CC01330E. 25848722

[r13] Chen H, Nwe PK, Yang Y, Rosen CE, Bielecka AA, Kuchroo M, Cline GW, Kruse AC, Ring AM, Crawford JM, et al. (2019) A forward chemical genetic screen reveals gut microbiota metabolites that modulate host physiology. Cell 177(5), 1217–1231.e1218. 10.1016/j.cell.2019.03.036. 31006530 PMC6536006

[r14] Chen PB, Black AS, Sobel AL, Zhao Y, Mukherjee P, Molparia B, Moore NE, Aleman Muench GR, Wu J, Chen W, et al. (2020) Directed remodeling of the mouse gut microbiome inhibits the development of atherosclerosis. Nature Biotechnology 38(11), 1288–1297. 10.1038/s41587-020-0549-5. PMC7641989 32541956

[r15] Cohen LJ, Kang HS, Chu J, Huang YH, Gordon EA, Reddy BV, Ternei MA, Craig JW and Brady SF (2015) Functional metagenomic discovery of bacterial effectors in the human microbiome and isolation of commendamide, a GPCR G2A/132 agonist. Proceedings of the National Academy of Sciences of the United States of America 112(35), E4825–4834. 10.1073/pnas.1508737112. 26283367 PMC4568208

[r16] Colosimo DA, Kohn JA, Luo PM, Piscotta FJ, Han SM, Pickard AJ, Rao A, Cross JR, Cohen LJ and Brady SF (2019) Mapping interactions of microbial metabolites with human g-protein-coupled receptors. Cell Host Microbe 26(2), 273–282.e277. 10.1016/j.chom.2019.07.002. 31378678 PMC6706627

[r17] Correia MSP, Jain A, Alotaibi W, Young Tie Yang P, Rodriguez-Mateos A and Globisch D (2020) Comparative dietary sulfated metabolome analysis reveals unknown metabolic interactions of the gut microbiome and the human host. Free Radical Biology and Medicine 160, 745–754. 10.1016/j.freeradbiomed.2020.09.006. 32927015

[r18] Coyne MJ, Zitomersky NL, McGuire AM, Earl AM and Comstock LE (2014) Evidence of extensive DNA transfer between bacteroidales species within the human gut. mBio 5(3), e01305–01314. 10.1128/mBio.01305-14. 24939888 PMC4073490

[r19] Croasdell A, Duffney PF, Kim N, Lacy SH, Sime PJ and Phipps RP (2015) PPARγ and the innate immune system mediate the resolution of inflammation. PPAR Res 2015, 549691. 10.1155/2015/549691. 26713087 PMC4680113

[r20] Cully M (2019) Microbiome therapeutics go small molecule. Nature Reviews Drug Discovery 18(8), 569–572. 10.1038/d41573-019-00122-8. 31367062

[r21] Das S, Angsantikul P, Le C, Bao D., Miyamoto Y, Gao W, Zhang L and Eckmann L (2018) Neutralization of cholera toxin with nanoparticle decoys for treatment of cholera. PLOS Neglected Tropical Diseases 12(2), e0006266. 10.1371/journal.pntd.0006266. 29470490 PMC5839590

[r22] Devlin AS and Fischbach MA (2015) A biosynthetic pathway for a prominent class of microbiota-derived bile acids. Nature Chemical Biology 11(9), 685–690. 10.1038/nchembio.1864. 26192599 PMC4543561

[r23] Di Paola M, Bonechi E, Provensi G, Costa A, Clarke G, Ballerini C, De Filippo C and Passani MB (2018) Oleoylethanolamide treatment affects gut microbiota composition and the expression of intestinal cytokines in Peyer’s patches of mice. Scientific Reports 8(1), 14881. 10.1038/s41598-018-32925-x. 30291258 PMC6173739

[r24] Diakite A, Dubourg G, Dione N, Afouda P, Bellali S, Ngom II, Valles C, Tall Ml, Lagier J-C and Raoult D (2020) Optimization and standardization of the culturomics technique for human microbiome exploration. Scientific Reports 10(1), 9674. 10.1038/s41598-020-66738-8. 32541790 PMC7295790

[r25] Ducarmon QR, Zwittink RD, Hornung BVH, van Schaik W, Young VB and Kuijper EJ (2019) Gut microbiota and colonization resistance against bacterial enteric infection. Microbiology and Molecular Biology Reviews 83(3), e0007–19. 10.1128/MMBR.00007-19. PMC6710460 31167904

[r26] Dvořák Z, Kopp F, Costello CM, Kemp JS, Li H, Vrzalová A, Štěpánková M, Bartoňková I, Jiskrová E, Poulíková K, et al. (2020) Targeting the pregnane X receptor using microbial metabolite mimicry. EMBO Molecular Medicine 12(4), e11621. 10.15252/emmm.201911621. 32153125 PMC7136958

[r27] Enam F and Mansell TJ (2019) Prebiotics: tools to manipulate the gut microbiome and metabolome. Journal of Industrial Microbiology and Biotechnology 46(9–10), 1445–1459. 10.1007/s10295-019-02203-4. 31201649

[r28] Fair RJ, Hahm HS and Seeberger PH (2015) Combination of automated solid-phase and enzymatic oligosaccharide synthesis provides access to α(2,3)-sialylated glycans. Chemical Communications 51(28), 6183–6185. 10.1039/C5CC01368B. 25754251

[r29] Fernandez-Lopez R, Machón C, Longshaw CM, Martin S, Molin S, Zechner EL, Espinosa M, Lanka E and de la Cruz F(2005) Unsaturated fatty acids are inhibitors of bacterial conjugation. Microbiology (Reading) 151(Pt 11), 3517–3526. 10.1099/mic.0.28216-0. 16272375

[r30] Forster ER, Yang X, Tai AK, Hang HC and Shen A (2022) Identification of a bile acid-binding transcription factor in *Clostridioides difficile* using chemical proteomics. ACS Chemical Biology 17(11), 3086–3099. 10.1021/acschembio.2c00463. 36279369 PMC10518218

[r31] Friedman ES, Li Y, Shen TD, Jiang J, Chau L, Adorini L, Babakhani F, Edwards J, Shapiro D, Zhao C, et al. (2018) FXR-dependent modulation of the human small intestinal microbiome by the bile acid derivative obeticholic acid. Gastroenterology 155(6), 1741–1752.e1745. 10.1053/j.gastro.2018.08.022. 30144429 PMC6279623

[r32] Garg N, Conway LP, Ballet C, Correia MSP, Olsson FKS, Vujasinovic M, Löhr JM and Globisch D (2018) Chemoselective probe containing a unique bioorthogonal cleavage site for investigation of gut microbiota metabolism. Angewandte Chemie International Edition 57(42), 138005–13809. 10.1002/anie.201804828. 30168889

[r33] Getino M, Fernández-López R, Palencia-Gándara C, Campos-Gómez J, Sánchez-López JM, Martínez M, Fernández A and de la Cruz F (2016) Tanzawaic acids, a chemically novel set of bacterial conjugation inhibitors. PLoS One 11(1), e0148098. 10.1371/journal.pone.0148098. 26812051 PMC4727781

[r34] Getino M, Sanabria-Ríos DJ, Fernández-López R, Campos-Gómez J, Sánchez-López JM, Fernández A, Carballeira NM and de la Cruz F (2015) Synthetic fatty acids prevent plasmid-mediated horizontal gene transfer. mBio 6(5), e01032–01015. 10.1128/mBio.01032-15. 26330514 PMC4556808

[r35] Geva-Zatorsky N, Alvarez D, Hudak JE, Reading NC, Erturk-Hasdemir D, Dasgupta S, von Andrian UH and Kasper DL (2015) In vivo imaging and tracking of host-microbiota interactions via metabolic labeling of gut anaerobic bacteria. Nature Medicine 21(9), 1091–1100. 10.1038/nm.3929. PMC4694768 26280120

[r36] Gibson GR, Probert HM, Loo JV, Rastall RA and Roberfroid MB (2004) Dietary modulation of the human colonic microbiota: updating the concept of prebiotics. Nutrition Research Reviews 17(2), 259–275. 10.1079/NRR200479. 19079930

[r37] Gil-Pichardo A, Sánchez-Ruiz A and Colmenarejo G (2023) Analysis of metabolites in human gut: illuminating the design of gut-targeted drugs. Journal of Cheminformatics 15, 96. 10.1186/s13321-023-00768-y. 37833792 PMC10571276

[r38] Goto T, Kim YI, Furuzono T, Takahashi N, Yamakuni K, Yang HE, Li Y, Ohue R, Nomura W, and Sugawara T, et al. (2015) 10-oxo-12(Z)-octadecenoic acid, a linoleic acid metabolite produced by gut lactic acid bacteria, potently activates PPARγ and stimulates adipogenesis. Biochemical and Biophysical Research Communications 459(4), 597–603. 10.1016/j.bbrc.2015.02.154. 25749343

[r39] Grabacka M, Pierzchalska M, Płonka PM and Pierzchalski P (2021) The role of PPAR alpha in the modulation of innate immunity. International Journal of Molecular Sciences 22(19), 10545. 10.3390/ijms221910545. 34638886 PMC8508635

[r40] Hatzenpichler R, Scheller S, Tavormina PL, Babin BM, Tirrell DA and Orphan VJ (2014) In situ visualization of newly synthesized proteins in environmental microbes using amino acid tagging and click chemistry. Environmental Microbiology 16(8), 2568–2590. 10.1111/1462-2920.12436. 24571640 PMC4122687

[r41] Hua S (2020) Advances in oral drug delivery for regional targeting in the gastrointestinal tract – influence of physiological, pathophysiological and pharmaceutical factors. Frontiers in Pharmacology 11, 524. 10.3389/fphar.2020.00524. 32425781 PMC7212533

[r42] Husted AS, Trauelsen M, Rudenko O, Hjorth SA and Schwartz TW (2017) GPCR-mediated signaling of metabolites. Cell Metabolism 25(4), 777–796. 10.1016/j.cmet.2017.03.008. 28380372

[r43] Iyer SS, Gensollen T, Gandhi A, Oh SF, Neves JF, Collin F, Lavin R, Serra C, Glickman J, de Silva PSA, et al. (2018) Dietary and microbial oxazoles induce intestinal inflammation by modulating aryl hydrocarbon receptor responses. Cell 173(5), 1123–1134.e1111. 10.1016/j.cell.2018.04.037. 29775592 PMC6119676

[r44] Joice R, Yasuda K, Shafquat A, Morgan XC and Huttenhower C (2014) Determining microbial products and identifying molecular targets in the human microbiome. Cell Metabolism 20(5), 731–741. 10.1016/j.cmet.2014.10.003. 25440055 PMC4254638

[r45] Jones BV, Begley M, Hill C, Gahan CG and Marchesi JR (2008) Functional and comparative metagenomic analysis of bile salt hydrolase activity in the human gut microbiome. Proceedings of the National Academy of Sciences of the United States of America 105(36), 13580–13585. 10.1073/pnas.0804437105. 18757757 PMC2533232

[r46] Jose S, Mukherjee A, Horrigan O, Setchell KDR, Zhang W, Moreno-Fernandez ME, Andersen H, Sharma D, Haslam DB, and Divanovic S, et al. (2021) Obeticholic acid ameliorates severity of Clostridioides difficile infection in high fat diet-induced obese mice. Mucosal Immunology 14(2), 500–510. 10.1038/s41385-020-00338-7. 32811993 PMC7889747

[r47] Joseph AA, Pardo-Vargas A and Seeberger PH (2020) Total synthesis of polysaccharides by automated glycan assembly. Journal of the American Chemical Society 142(19), 8561–8564. 10.1021/jacs.0c00751. 32338884 PMC7304863

[r48] Kang JD, Myers CJ, Harris SC, Kakiyama G, Lee IK, Yun BS, Matsuzaki K, Furukawa M, Min HK, Bajaj JS, et al. (2019) Bile acid 7α-dehydroxylating gut bacteria secrete antibiotics that inhibit clostridium difficile: role of secondary bile acids. Cell Chemical Biology 26(1), 27–34.e24. 10.1016/j.chembiol.2018.10.003. 30482679 PMC6338514

[r49] Katkar GD, Sayed IM, Anandachar MS, Castillo V, Vidales E, Toobian D, Usmani F, Sawires JR, Leriche G, Yang J, et al. (2022) Artificial intelligence-rationalized balanced PPARα/γ dual agonism resets dysregulated macrophage processes in inflammatory bowel disease. Communications Biology 5(1), 231. 10.1038/s42003-022-03168-4. 35288651 PMC8921270

[r50] Khodakivskyi PV, Lauber CL, Yevtodiyenko A, Bazhin AA, Bruce S, Ringel-Kulka T, Ringel Y, Bétrisey B, Torres J, Hu J, et al. (2021) Noninvasive imaging and quantification of bile salt hydrolase activity: From bacteria to humans. Science Advances 7(6), eaaz9857. 10.1126/sciadv.aaz9857. 33536224 PMC7857686

[r51] Kovatcheva-Datchary P, Shoaie S, Lee S, Wahlström A, Nookaew I, Hallen A, Perkins R, Nielsen J and Bäckhed F (2019) Simplified intestinal microbiota to study microbe-diet-host interactions in a mouse model. Cell Reports 26(13), 3772–3783.e3776. 10.1016/j.celrep.2019.02.090. 30917328 PMC6444000

[r52] Kraehenbuhl JP and Neutra MR (1992) Molecular and cellular basis of immune protection of mucosal surfaces. Physiological Reviews 72(4), 853–879. 10.1152/physrev.1992.72.4.853. 1438580

[r53] Kroeze WK, Sassano MF, Huang XP, Lansu K, McCorvy JD, Giguère PM, Sciaky N and Roth BL (2015) PRESTO-Tango as an open-source resource for interrogation of the druggable human GPCRome. Nature Structural & Molecular Biology 22(5), 362–369. 10.1038/nsmb.3014. PMC4424118 25895059

[r54] Lamas B, Hernandez-Galan L, Galipeau H. J, Constante M, Clarizio A, Jury J, Breyner NM, Caminero A, Rueda G, Hayes CL, et al. (2020) Aryl hydrocarbon receptor ligand production by the gut microbiota is decreased in celiac disease leading to intestinal inflammation. Science Translational Medicine 12(566), eaba0624. 10.1126/scitranslmed.aba0624. 33087499

[r55] Laucirica DR, Triantis V, Schoemaker R, Estes MK and Ramani S (2017) Milk oligosaccharides inhibit human rotavirus infectivity in MA104 cells. Journal of Nutrition 147(9), 1709–1714. 10.3945/jn.116.246090. 28637685 PMC5572490

[r57] Lee MT, Le HH and Johnson EL (2021) Dietary sphinganine is selectively assimilated by members of the mammalian gut microbiome. Journal of Lipid Research 62, 100034. 10.1194/jlr.RA120000950. 32646940 PMC7910519

[r58] Lefebvre P, Cariou B, Lien F, Kuipers F and Staels B (2009) Role of bile acids and bile acid receptors in metabolic regulation. Physiological Reviews 89(1), 147–191. 10.1152/physrev.00010.2008. 19126757

[r59] Li F, Jiang C, Krausz KW, Li Y, Albert I, Hao H, Fabre KM, Mitchell JB, Patterson AD and Gonzalez FJ (2013) Microbiome remodelling leads to inhibition of intestinal farnesoid X receptor signalling and decreased obesity. Nature Communications 4, 2384. 10.1038/ncomms3384. PMC6595219 24064762

[r60] Li T and Chiang JY (2014) Bile acid signaling in metabolic disease and drug therapy. Pharmacological Reviews 66(4), 948–983. 10.1124/pr.113.008201. 25073467 PMC4180336

[r61] Li W, McArthur JB and Chen X (2019) Strategies for chemoenzymatic synthesis of carbohydrates. Carbohydrate Research 472, 86–97. 10.1016/j.carres.2018.11.014. 30529493 PMC6342554

[r62] Li Z, McCafferty KJ and Judd RL (2021) Role of HCA_2_ in regulating intestinal homeostasis and suppressing colon carcinogenesis. Frontiers in Immunology 12, 606384. 10.3389/fimmu.2021.606384. 33708203 PMC7940178

[r63] Lin L, Wu Q, Song J, Du Y, Gao J, Song Y, Wang W and Yang C (2020) Revealing the in vivo growth and division patterns of mouse gut bacteria. Science Advances 6(36). 10.1126/sciadv.abb2531. PMC7473744 32917613

[r64] Lin S, Zhang Z, Xu H, Li L, Chen S, Li J, Hao Z and Chen PR (2011) Site-specific incorporation of photo-cross-linker and bioorthogonal amino acids into enteric bacterial pathogens. Journal of the American Chemical Society 133(50), 20581–20587. 10.1021/ja209008w. 22084898

[r65] Lin YH, Luck H, Khan S, Schneeberger PHH, Tsai S, Clemente-Casares X, Lei H, Leu YL, Chan YT, Chen HY, et al. (2019) Aryl hydrocarbon receptor agonist indigo protects against obesity-related insulin resistance through modulation of intestinal and metabolic tissue immunity. International Journal of Obesity 43(12), 2407–2421. 10.1038/s41366-019-0340-1. 30944419 PMC6892742

[r66] Liu J, Xu Q, Zhang J, Zhou X, Lyu F, Zhao P and Ding Y (2015) Preparation, composition analysis and antioxidant activities of konjac oligo-glucomannan. Carbohydrate Polymers 130, 398–404. 10.1016/j.carbpol.2015.05.025. 26076641

[r67] Liu P, Hu S, He Z, Feng C, Dong G, An S, Liu R, Xu F, Chen Y and Ying X (2022a) Towards strain-level complexity: sequencing depth required for comprehensive single-nucleotide polymorphism analysis of the human gut microbiome. Frontiers in Microbiology 13, 828254. 10.3389/fmicb.2022.828254. 35602026 PMC9119422

[r68] Liu Q, Tian X, Maruyama D, Arjomandi M and Prakash A (2021) Lung immune tone via gut-lung axis: gut-derived LPS and short-chain fatty acids’ immunometabolic regulation of lung IL-1β, FFAR2, and FFAR3 expression. American Journal of Physiology Lung Cellular and Molecular Physiology 321(1), L65–L78. 10.1152/ajplung.00421.2020. 33851870 PMC8321849

[r69] Liu S, Moon CD, Zheng N, Huws S, Zhao S and Wang J (2022b) Opportunities and challenges of using metagenomic data to bring uncultured microbes into cultivation. Microbiome 10(1), 76. 10.1186/s40168-022-01272-5. 35546409 PMC9097414

[r70] Ltd., I. U. (2018) *Microbiome Modulator Drugs – The New Generation of Therapeutic.* https://www.scribd.com/document/513306656/Pharmaprojects-Microbiome-Whitepaper.

[r71] Ma N, He T, Johnston LJ and Ma X (2020) Host-microbiome interactions: the aryl hydrocarbon receptor as a critical node in tryptophan metabolites to brain signaling. Gut Microbes 11(5), 1203–1219. 10.1080/19490976.2020.1758008. 32401136 PMC7524279

[r72] Malwe AS, Srivastava GN and Sharma VK (2023) GutBug: A tool for prediction of human gut bacteria mediated biotransformation of biotic and xenobiotic molecules using machine learning. Journal of Molecular Biology 435(14), 168056. 10.1016/j.jmb.2023.168056. 37356904

[r73] Mayers MD, Moon C, Stupp GS, Su AI and Wolan DW (2017) Quantitative metaproteomics and activity-based probe enrichment reveals significant alterations in protein expression from a mouse model of inflammatory bowel disease. Journal of Proteome Research 16(2), 1014–1026. 10.1021/acs.jproteome.6b00938. 28052195 PMC5441882

[r74] Munck C, Sheth RU, Freedberg DE and Wang HH (2020) Recording mobile DNA in the gut microbiota using an Escherichia coli CRISPR-Cas spacer acquisition platform. Nature Communications 11(1), 95. 10.1038/s41467-019-14012-5. PMC6946703 31911609

[r75] Muroski JM, Fu JY, Nguyen HH, Wofford NQ, Mouttaki H, James KL, McInerney MJ, Gunsalus RP, Loo JA and Ogorzalek Loo RR (2022) The acyl-proteome of syntrophus aciditrophicus reveals metabolic relationships in benzoate degradation. Molecular & Cellular Proteomics 21(4), 100215. 10.1016/j.mcpro.2022.100215. 35189333 PMC8942843

[r76] Ndeh D, Rogowski A, Cartmell A, Luis AS, Baslé A, Gray J, Venditto I, Briggs J, Zhang X, Labourel A, et al. (2017) Complex pectin metabolism by gut bacteria reveals novel catalytic functions. Nature 544(7648), 65–70. 10.1038/nature21725. 28329766 PMC5388186

[r77] Negatu DA, Gengenbacher M, Dartois V and Dick T (2020) Indole propionic acid, an unusual antibiotic produced by the gut microbiota, with anti-inflammatory and antioxidant properties. Frontiers in Microbiology 11, 575586. 10.3389/fmicb.2020.575586. 33193190 PMC7652848

[r78] Negatu DA, Liu JJJ, Zimmerman M, Kaya F, Dartois V, Aldrich CC, Gengenbacher M and Dick T (2018) Whole-cell screen of fragment library identifies gut microbiota metabolite indole propionic acid as antitubercular. Antimicrobial Agents and Chemotherapy 62(3), e01571–17. 10.1128/aac.01571-17. 29229639 PMC5826148

[r79] Newman MA, Petri RM, Grüll D, Zebeli Q and Metzler-Zebeli BU (2018) Transglycosylated starch modulates the gut microbiome and expression of genes related to lipid synthesis in liver and adipose tissue of pigs. Frontiers In Microbiology 9, 224. 10.3389/fmicb.2018.00224. 29487593 PMC5816791

[r80] Nie Q, Luo X, Wang K, Ding Y, Jia S, Zhao Q, Li M et al (2024) Cell 187(11), 2717–2734.e33. 10.1016/j.cell.2024.03.034. 38653239

[r81] Nilsson NE, Kotarsky K, Owman C and Olde B (2003) Identification of a free fatty acid receptor, FFA2R, expressed on leukocytes and activated by short-chain fatty acids. Biochemical and Biophysical Research Communications 303(4), 1047–1052. 10.1016/s0006-291x(03)00488-1. 12684041

[r82] Offermanns S (2017) Hydroxy-carboxylic acid receptor actions in metabolism. Trends in Endocrinology & Metabolism 28(3), 227–236. 10.1016/j.tem.2016.11.007. 28087125

[r83] Orman M, Bodea S, Funk MA, Campo AM, Bollenbach M, Drennan CL and Balskus EP (2019) Structure-guided identification of a small molecule that inhibits anaerobic choline metabolism by human gut bacteria. Journal of the American Chemical Society 141(1), 33–37. 10.1021/jacs.8b04883. 30557011 PMC6475491

[r84] Oyedemi BO, Shinde V, Shinde K, Kakalou D, Stapleton PD and Gibbons S (2016) Novel R-plasmid conjugal transfer inhibitory and antibacterial activities of phenolic compounds from Mallotus philippensis (Lam.) Mull. Arg. Journal of Global Antimicrobial Resistance 5, 15–21. 10.1016/j.jgar.2016.01.011. 27436460

[r85] Palencia-Gándara C, Getino M, Moyano G, Redondo S, Fernández-López R, González-Zorn B and de la Cruz F (2021) Conjugation inhibitors effectively prevent plasmid transmission in natural environments. mBio 12(4), e0127721. 10.1128/mBio.01277-21. 34425705 PMC8406284

[r86] Parasar B, Zhou H, Xiao X, Shi Q, Brito IL and Chang PV (2019) Chemoproteomic profiling of gut microbiota-associated bile salt hydrolase activity. ACS Central Science 5(5), 867–873. 10.1021/acscentsci.9b00147. 31139722 PMC6535767

[r87] Patangia DV, Anthony Ryan C, Dempsey E, Paul Ross R and Stanton C (2022) Impact of antibiotics on the human microbiome and consequences for host health. Microbiologyopen 11(1), e1260. 10.1002/mbo3.1260. 35212478 PMC8756738

[r88] Pellock SJ, Creekmore BC, Walton WG, Mehta N, Biernat KA, Cesmat AP, Ariyarathna Y, Dunn ZD, Li B, Jin J, et al. (2018) Gut microbial β-glucuronidase inhibition via catalytic cycle interception. ACS Central Science 4(7), 868–879. 10.1021/acscentsci.8b00239. 30062115 PMC6062831

[r89] Peters A, Krumbholz P, Jäger E, Heintz-Buschart A, Çakir MV, Rothemund S, Gaudl A, Ceglarek U, Schöneberg T and Stäubert C (2019) Metabolites of lactic acid bacteria present in fermented foods are highly potent agonists of human hydroxycarboxylic acid receptor 3. PLOS Genetics 15(5), e1008145. 10.1371/journal.pgen.1008145. 31120900 PMC6532841

[r90] Poletto P, Pereira GN, Monteiro CRM, Pereira MAF, Bordignon SE and de Oliveira D (2020) Xylooligosaccharides: Transforming the lignocellulosic biomasses into valuable 5-carbon sugar prebiotics. Process Biochemistry 91, 352–363. 10.1016/j.procbio.2020.01.005.

[r91] Priyadarshini M, Lednovich K, Xu K, Gough S, Wicksteed B and Layden BT (2021) FFAR from the gut microbiome crowd: SCFA receptors in T1D pathology. Metabolites 11(5), 302. 10.3390/metabo11050302. 34064625 PMC8151283

[r92] Priyadarshini M, Navarro G and Layden BT (2018) Gut microbiota: FFAR reaching effects on islets. Endocrinology 159(6), 2495–2505. 10.1210/en.2018-00296. 29846565 PMC6692871

[r93] Pulakazhi Venu VK, Saifeddine M, Mihara K, Tsai YC, Nieves K, Alston L, Mani S, McCoy KD, Hollenberg MD and Hirota SA (2019) The pregnane X receptor and its microbiota-derived ligand indole 3-propionic acid regulate endothelium-dependent vasodilation. American Journal of Physiology Endocrinology and Metabolism 317(2), E350–E361. 10.1152/ajpendo.00572.2018. 31211619 PMC6732469

[r94] Putnam EE, Abellon-Ruiz J, Killinger BJ, Rosnow JJ, Wexler AG, Folta-Stogniew E, Wright AT, van den Berg B and Goodman AL (2022) Gut commensal *Bacteroidetes* encode a novel class of vitamin B_12_-Binding Proteins. mBio 13, e02845–21. 10.1128/mbio.02845-21. 35227073 PMC8941943

[r95] Quinn RA, Melnik AV, Vrbanac A, Fu T, Patras KA, Christy MP, Bodai Z, Belda-Ferre P, Tripathi A and Chung LK, et al. (2020) Global chemical effects of the microbiome include new bile-acid conjugations. Nature 579(7797), 123–129. 10.1038/s41586-020-2047-9. 32103176 PMC7252668

[r96] Rackaityte E and Lynch SV (2020) The human microbiome in the 21st century. Nature Communications 11(1), 5256. 10.1038/s41467-020-18983-8. PMC7567807 33067429

[r97] Randall TD and Mebius RE (2014) The development and function of mucosal lymphoid tissues: a balancing act with micro-organisms. Mucosal Immunology 7(3), 455–466. 10.1038/mi.2014.11. 24569801

[r98] Rea MC, Sit CS, Clayton E, O’Connor PM, Whittal RM, Zheng J, Vederas JC, Ross RP and Hill C. (2010) Thuricin CD, a posttranslationally modified bacteriocin with a narrow spectrum of activity against Clostridium difficile. Proceedings of the National Academy of Sciences of the United States of America 107(20), 9352–9357. 10.1073/pnas.0913554107. 20435915 PMC2889069

[r99] Ripoll-Rozada J, García-Cazorla Y, Getino M., Machón C, Sanabria-Ríos D, de la Cruz F, Cabezón E and Arechaga I (2016) Type IV traffic ATPase TrwD as molecular target to inhibit bacterial conjugation. Molecular Microbiology 100(5), 912–921. 10.1111/mmi.13359. 26915347 PMC4908816

[r100] Ruiz-Palacios GM, Cervantes LE, Ramos P, Chavez-Munguia B and Newburg DS (2003) Campylobacter jejuni binds intestinal H(O) antigen (Fuc alpha 1, 2Gal beta 1, 4GlcNAc), and fucosyloligosaccharides of human milk inhibit its binding and infection. Journal of Biological Chemistry 278(16), 14112–14120. 10.1074/jbc.M207744200. 12562767

[r101] Santilli AD, Dawson EM, Whitehead KJ and Whitehead DC (2018) Nonmicrobicidal small molecule inhibition of polysaccharide metabolism in human gut microbes: a potential therapeutic avenue. ACS Chemical Biology 13(5), 1165–1172. 10.1021/acschembio.8b00309. 29660284

[r102] Savidge T and Sorg JA (2019) Role of bile in infectious disease: the gall of 7α-dehydroxylating gut bacteria. Cell Chemical Biology 26(1), 1–3. 10.1016/j.chembiol.2018.12.010. 30658109 PMC6542261

[r103] Schroeder BO and Bäckhed F (2016) Signals from the gut microbiota to distant organs in physiology and disease. Nature Medicine 22(10), 1079–1089. 10.1038/nm.4185. 27711063

[r104] Shelton AN, Seth EC, Mok KC, Han AW, Jackson SN, Haft DR and Taga ME (2019) Uneven distribution of cobamide biosynthesis and dependence in bacteria predicted by comparative genomics. The ISME Journal 13(3), 789–804. 10.1038/s41396-018-0304-9. 30429574 PMC6461909

[r105] Shinde R and McGaha TL (2018) The aryl hydrocarbon receptor: connecting immunity to the microenvironment. Trends in Immunology 39(12), 1005–1020. 10.1016/j.it.2018.10.010. 30409559 PMC7182078

[r106] Shreiner AB, Kao JY and Young VB (2015) The gut microbiome in health and in disease. Current Opinion in Gastroenterology 31(1), 69–75. 10.1097/MOG.0000000000000139. 25394236 PMC4290017

[r107] Silva-Veiga FM, Miranda CS, Martins FF, Daleprane JB, Mandarim-de-Lacerda CA and Souza-Mello V (2020) Gut-liver axis modulation in fructose-fed mice: a role for PPAR-alpha and linagliptin. Journal of Endocrinology 247(1), 11–24. 10.1530/JOE-20-0139. 32698143

[r108] Silva-Veiga FM, Miranda CS, Vasques-Monteiro IML, Souza-Tavares H, Martins FF, Daleprane JB and Souza-Mello V (2022) Peroxisome proliferator-activated receptor-alpha activation and dipeptidyl peptidase-4 inhibition target dysbiosis to treat fatty liver in obese mice. World Journal of Gastroenterology 28(17), 1814–1829. 10.3748/wjg.v28.i17.1814. 35633911 PMC9099201

[r109] Sorbara MT and Pamer EG (2019) Interbacterial mechanisms of colonization resistance and the strategies pathogens use to overcome them. Mucosal Immunology 12(1), 1–9. 10.1038/s41385-018-0053-0. 29988120 PMC6312114

[r110] Spaulding CN, Klein RD, Ruer S, Kau AL, Schreiber HL, Cusumano ZT, Dodson KW, Pinkner JS, Fremont DH, Janetka JW, et al. (2017) Selective depletion of uropathogenic E. coli from the gut by a FimH antagonist. Nature 546(7659), 528–532. 10.1038/nature22972. 28614296 PMC5654549

[r111] Spaulding CN, Klein RD, Schreiber HL, Janetka JW and Hultgren SJ (2018) Precision antimicrobial therapeutics: the path of least resistance? npj Biofilms and Microbiomes 4(1), 4. 10.1038/s41522-018-0048-3. 29507749 PMC5829159

[r112] Stecher B, Denzler R, Maier L, Bernet F, Sanders MJ, Pickard DJ, Barthel M, Westendorf AM, Krogfelt KA, Walker AW, et al. (2012) Gut inflammation can boost horizontal gene transfer between pathogenic and commensal Enterobacteriaceae. Proceedings of the National Academy of Sciences of the United States of America 109(4), 1269–1274. 10.1073/pnas.1113246109. 22232693 PMC3268327

[r113] Subramanian S, Huq S, Yatsunenko T, Haque R, Mahfuz M, Alam MA, Benezra A, DeStefano J, Meier MF, Muegge BD, et al. (2014) Persistent gut microbiota immaturity in malnourished Bangladeshi children. Nature 510(7505), 417–421. 10.1038/nature13421. 24896187 PMC4189846

[r114] Trikha P and Lee DA (2020) The role of AhR in transcriptional regulation of immune cell development and function. Biochimica et Biophysica Acta (BBA) – Reviews on Cancer 1873(1), 188335. 10.1016/j.bbcan.2019.188335. 31816350

[r115] Turnbaugh PJ, Ley RE, Hamady M, Fraser-Liggett CM, Knight R and Gordon JI (2007) The human microbiome project. Nature 449(7164), 804–810. 10.1038/nature06244. 17943116 PMC3709439

[r116] Vohra Y, Vasan M, Venot A and Boons GJ (2008) One-pot synthesis of oligosaccharides by combining reductive openings of benzylidene acetals and glycosylations. Organic Letters 10(15), 3247–3250. 10.1021/ol801076w. 18578494 PMC2814312

[r117] Wang B, Yao M, Lv L, Ling Z and Li L (2017) The human microbiota in health and disease. Engineering 3(1), 71–82. 10.1016/J.ENG.2017.01.008.

[r118] Wang S, Lei T, Zhang K, Zhao W, Fang L, Lai B, Han J, Xiao L and Wang N (2014) Xenobiotic pregnane X receptor (PXR) regulates innate immunity via activation of NLRP3 inflammasome in vascular endothelial cells. Journal of Biological Chemistry 289(43), 30075–30081. 10.1074/jbc.M114.578781. 25202020 PMC4208014

[r119] Wang W, Lin L, Du Y, Song Y, Peng X, Chen X and Yang CJ (2019) Assessing the viability of transplanted gut microbiota by sequential tagging with D-amino acid-based metabolic probes. Nature Communications 10(1), 1317. 10.1038/s41467-019-09267-x. PMC6428874 30899006

[r120] Wang X, Yu C, Liu X, Yang J, Feng Y, Wu Y, Xu Y, Zhu Y and Li W (2022) Fenofibrate ameliorated systemic and retinal inflammation and modulated gut microbiota in high-fat diet-induced mice. Frontiers in Cellular and Infection Microbiology 12, 839592. 10.3389/fcimb.2022.839592. 35719341 PMC9201033

[r121] Wu L, Han Y, Zheng Z, Zhu S, Chen J, Yao Y, Yue S, Teufel A, Weng H, Li L, et al. (2021) Obeticholic acid inhibits anxiety via alleviating gut microbiota-mediated microglia accumulation in the brain of high-fat high-sugar diet mice. Nutrients 13(3), 940. 10.3390/nu13030940. 33803974 PMC7999854

[r122] Yang X, Stein KR and Hang HC (2023) Anti-infective bile acids bind and inactivate a Salmonella virulence regulator. Nature Chemical Biology 19, 91–100. 10.1038/s41589-022-01122-3. 36175659 PMC9805502

[r123] Zelante T, Iannitti RG, Cunha C, De Luca A, Giovannini G, Pieraccini G, Zecchi R, D’Angelo C, Massi-Benedetti C, Fallarino F, et al. (2013) Tryptophan catabolites from microbiota engage aryl hydrocarbon receptor and balance mucosal reactivity via interleukin-22. Immunity 39(2), 372–385. 10.1016/j.immuni.2013.08.003. 23973224

[r124] Zhang L, Xie C, Nichols RG, Chan S H, Jiang C, Hao R, Smith PB, Cai J, Simons MN, Hatzakis E, et al. (2016) Farnesoid X receptor signaling shapes the gut microbiota and controls hepatic lipid metabolism. mSystems 1(5), e00070–16. 10.1128/mSystems.00070-16. 27822554 PMC5080402

[r125] Zhang ZJ, Wang YC, Yang X and Hang HC (2020) Chemical reporters for exploring microbiology and microbiota mechanisms. Chembiochem 21(1–2), 19–32. 10.1002/cbic.201900535. 31730246 PMC7008969

[r126] Zheng J, Li H, Zhang X, Jiang M, Luo C, Lu Z, Xu Z and Shi J (2018) Prebiotic mannan-oligosaccharides augment the hypoglycemic effects of metformin in correlation with modulating gut microbiota. Journal of Agricultural and Food Chemistry 66(23), 5821–5831. 10.1021/acs.jafc.8b00829. 29701959

[r127] Zhu H, Chen Z, Ma Z, Tan H, Xiao C, Tang X, Zhang B, Wang Y and Gao Y (2017) Tanshinone IIA protects endothelial cells from H₂O₂-induced injuries via PXR activation. Biomolecules & Therapeutics (Seoul) 25(6), 599–608. 10.4062/biomolther.2016.179. PMC5685429 28173640

[r128] Zhu W, Winter MG, Byndloss MX, Spiga L, Duerkop BA, Hughes ER, Büttner L, de Lima Romão E, Behrendt CL, Lopez CA, et al. (2018) Precision editing of the gut microbiota ameliorates colitis. Nature 553(7687), 208–211. 10.1038/nature25172. 29323293 PMC5804340

[r129] Zimmermann M., Zimmermann-Kogadeeva M, Wegmann R and Goodman AL (2019) Separating host and microbiome contributions to drug pharmacokinetics and toxicity. Science 363(6427), eaat9931. 10.1126/science.aat9931. 30733391 PMC6533120

